# Unveiling Mixed Apical Hypertrophic Cardiomyopathy: A Case Study on Hypovolemia-Induced Syncope

**DOI:** 10.7759/cureus.81646

**Published:** 2025-04-03

**Authors:** Ethan Miller, Marjorie Cadestin, Parita Patel

**Affiliations:** 1 Internal Medicine, Cooper Medical School of Rowan University, Camden, USA; 2 Internal Medicine, Cooper University Hospital, Camden, USA

**Keywords:** diastolic dysfunction, hypovolemia, left ventricular outflow obstruction, mixed apical hypertrophic cardiomyopathy, syncope

## Abstract

Mixed apical hypertrophic cardiomyopathy (MAHCM) is a rare variant of hypertrophic cardiomyopathy characterized by hypertrophy of the left ventricular (LV) apex with septal involvement, increasing the risk of left ventricular outflow tract (LVOT) obstruction, mid-ventricular obstruction, and adverse cardiovascular events such as syncope and sudden cardiac death. We describe an 84-year-old female with a history of hypertension and hyperlipidemia who presented with a syncopal episode preceded by dizziness, blurred vision, and diaphoresis, with a history of poor oral intake and weight loss. She was hemodynamically stable but exhibited orthostatic changes, and laboratory findings revealed acute kidney injury (AKI) suggesting hypovolemia. Electrocardiogram (ECG) showed normal sinus rhythm with left ventricular hypertrophy and repolarization abnormalities, while transthoracic echocardiography (TTE) revealed a hyperdynamic left ventricular ejection fraction (LVEF) of 75%, LV apical hypertrophy with septal involvement, mild systolic anterior motion of the mitral valve leaflet with trace mitral regurgitation, and diastolic dysfunction. The measured LVOT diameter, E/A ratio, E/E' ratio, and septal E' velocity per TTE indicated LVOT obstruction and diastolic dysfunction. The patient was treated with intravenous fluids for hypovolemia and initiated on carvedilol, leading to symptom resolution. Her clinical presentation, ECG findings, and echocardiographic parameters were suggestive of MAHCM, though differentiation from hypertensive heart disease remained a consideration. This case highlights the role of dehydration-induced hypovolemia in precipitating syncope in patients with MAHCM and underscores the importance of recognizing MAHCM in patients presenting with unexplained syncope. Early identification and management are critical in preventing complications such as arrhythmias and sudden cardiac death. Given the demographic variability and under-recognized nature of MAHCM, clinicians should maintain a high index of suspicion in elderly patients with hypertension who exhibit syncope, particularly in the setting of hypovolemia.

## Introduction

Mixed apical hypertrophic cardiomyopathy (MAHCM) is a rare subtype of hypertrophic cardiomyopathy characterized by predominant apical involvement with septal hypertrophy. It is diagnosed by cardiac imaging with increased apical and ventricular septal wall thickness, and electrocardiogram with tall R waves and precordial T wave inversions. Specifically, left ventricular (LV) hypertrophy predominating in the LV apex, with the apical wall thickness ≥ 15 mm and a ratio of maximal apical to posterior wall thickness ≥ 1.5, based on echocardiography supports the diagnosis [1]. The manifestations of MAHCM differ from pure apical HCM (ApHCM) due to an elevated risk of left ventricular outflow tract (LVOT) obstruction and mid-ventricular obstruction and cavity obliteration [2]. Its clinical presentation ranges from asymptomatic to dyspnea and syncope, to apical aneurysms, VT arrhythmias, and sudden cardiac death. In a study of 150 ApHCM patients, 59.4% had hypertrophy extending beyond the apex, indicating a mixed phenotype [3]. MAHCM shows significant demographic variations. Black patients and hypertensive male patients are more likely to present with the mixed phenotype compared to White, Asian, and non-hypertensive male patients. Females with MAHCM tend to be diagnosed at an older age and exhibit less prominent ECG changes. Hypertension is a significant environmental risk factor for MAHCM. Other factors such as obesity and physical activity levels may also influence the clinical expression and severity of the disease [4]. Genetic mutations in sarcomere proteins, such as MYBPC3 and MYH7, are implicated in HCM, including its apical and mixed forms. Specific mutations, such as the cardiac actin Glu101Lys, have been associated with apical hypertrophy. The genetic basis of MAHCM suggests interactions among genetic etiology, background modifier genes, and hemodynamic factors [5]. In this case, we present a patient with new-onset syncope in the setting of hypovolemia secondary to dehydration, found to have echocardiographic imaging and ECG findings suggestive of MAHCM.

## Case presentation

An 84-year-old African American female with a past medical history of hypertension and hyperlipidemia presented to the hospital with dizziness followed by a syncopal episode. The patient reported an acute onset of blurred vision, followed by dizziness, diaphoresis, and loss of consciousness while resting in a seated position. The patient regained consciousness in about 3 minutes and did not experience urinary or fecal incontinence. There was no postictal state reported. The patient recalled drinking coffee prior to the event, but no water. She reports generally having lost a considerable amount of weight over the past year accompanied by poor intake of food and hydrating fluids. She presented hemodynamically stable and the physical exam was remarkable for orthostatic hypotension. Relevant lab values are listed in Table 1. Notably, the previous creatinine baseline was 0.8mg/dL.

**Table 1:** Vitals and lab values including blood pressure (BP), pulse, respiratory rate (RR), body temperature (temp), SpO2, and serum creatinine

BP	Pulse	RR	Temp	SpO2	Creatinine
113/62	83/min	18/min	97.7 °F	100%	1.21 mg/dL

**Figure 1:**
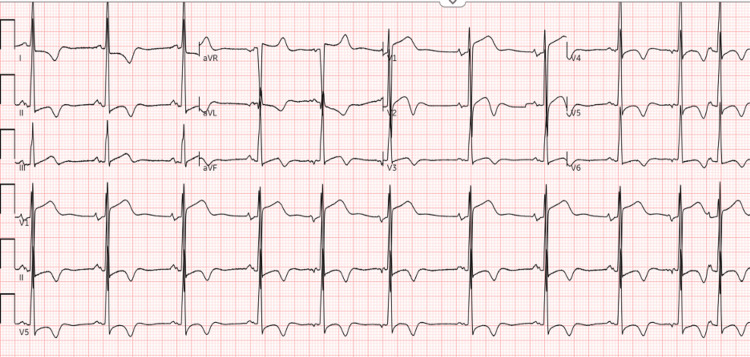
ECG showing sinus rhythm with premature atrial complexes, ST segment abnormalities in inferior leads and biphasic T waves in septal leads, and left ventricular hypertrophy with repolarization abnormality.

ECG revealed normal sinus rhythm with premature atrial complexes and left ventricular hypertrophy with repolarization abnormality (Figure 1).

**Figure 2:**
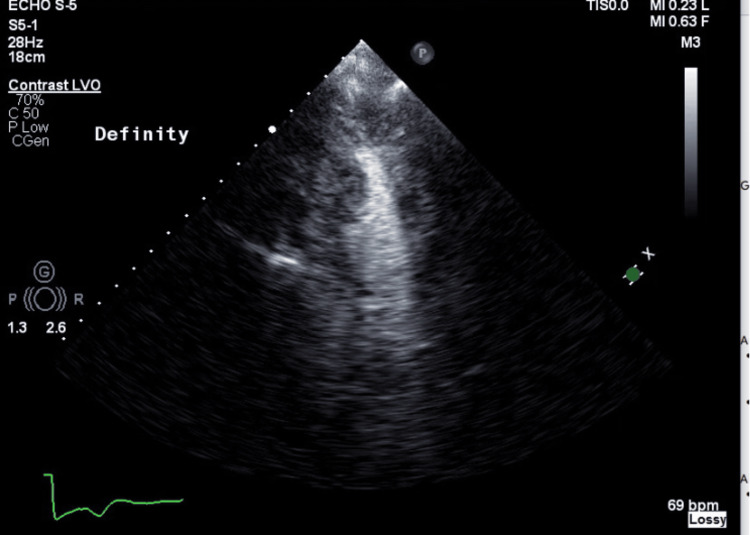
Echocardiogram showing apical four chamber view. In view is the left ventricle with mildly increased left ventricular wall thickness at the base with more prominent hypertrophy in the apical segments.

Transthoracic echocardiography (TTE) revealed hyperdynamic left ventricular ejection fraction of 75%, left ventricular apical wall and left ventricular wall thickening consistent with MAHCM, mildly thickened mitral valve with trace mitral regurgitation and mild systolic anterior motion (Figure 2). Abnormalities in TTE measurements are as follows (Table 2) [6].

**Table 2:** Echocardiogram measurement abnormalities, including left ventricular outflow tract (LVOT) diameter, mitral valve E/A ratio, mitral valve E/E' ratio, and septal E' velocity

LVOT Diameter	Mitral Valve E/A Ratio	Mitral Valve E/E' Ratio	Septal E' Velocity
1.6 cm	0.68	12.16	4.25 cm/s

ECG and TTE were suggestive of MAHCM. She was subsequently treated with intravenous fluids and initiated on carvedilol with improvement in her symptoms.

## Discussion

This case emphasizes the need to evaluate for structural cardiomyopathies in patients presenting with unexplained new-onset syncopal symptoms, especially in the setting of history of predisposing factors such as chronic hypertension. In the case of our patient, her history of reduced fluid intake in addition to mild AKI suggested by a rise in serum creatinine from 0.8 mg/dL baseline to 1.21 mg/dL, orthostatic hypotension and syncopal episode led to suspicion of hypovolemia. While her presenting symptoms could have been attributed to vasovagal symptoms in the setting of dehydration, her ECG and TTE had findings suggestive of MAHCM. Research has shown that patients with HCM may present with repolarization abnormalities such as non-specific ST-T changes on ECG [7]. For MAHCM, imaging demonstrates hypertrophy of the apex and interventricular septum, leading to more pronounced symptoms due to LVOTO. Specifically for our patient, the echocardiogram reveals abnormalities in the LVOT diameter, mitral valve E/A ratio, E/E' ratio, and septal E' velocity, suggesting diastolic dysfunction and a smaller than normal LVOT. The LVOT measurement of 1.6 cm is below the normal range of 1.8 to 2.5 cm. The E/A ratio of 0.68 is indicative of impaired relaxation, which is a sign of diastolic dysfunction. Normally, the E/A ratio should be between 1 and 2 in adults. The E/E' ratio of 12.16 suggests elevated left atrial pressure, which is consistent with diastolic dysfunction. The septal E' velocity of 4.25 cm/s is lower than the normal range (>8 cm/s), indicating impaired myocardial relaxation (Table 2) [6].

Our proposed mechanism for dehydration-induced hypovolemia representing a risk factor for syncope in a patient with HCM with diastolic dysfunction and a small LVOT is as follows: Hypovolemia leads to a decrease in circulating blood volume, which in turn reduces venous return to the heart. This diminished venous return results in decreased preload, limiting the LV end-diastolic volume. In a patient with underlying diastolic dysfunction, as indicated by a low E/A ratio (0.68) and elevated E/E' ratio (12.16), the ability of the LV to relax and fill adequately is compromised. The reduced preload further exacerbates this issue by decreasing stroke volume and cardiac output. Additionally, in conditions where dynamic left ventricular outflow tract obstruction (LVOTO) is present, such as in patients with a smaller than normal LVOT diameter (1.6 cm), the reduced preload can worsen obstruction due to inadequate LV diastolic function [8]. The combination of diastolic dysfunction and dynamic LVOTO ultimately leads to inadequate systemic perfusion, resulting in transient loss of consciousness or syncopal episodes when perfusion to the brain is limited. It is important to note that the findings reported in this case in corroboration with clinical faculty were suggestive of MAHCM and did not elicit a definitive diagnosis. Further investigation would need to be considered to differentiate MAHCM from conditions such as hypertensive heart disease in which this patient is also at risk for based on her past medical history.

## Conclusions

This case underscores the importance of recognizing hypovolemia as a precipitating factor for syncope in patients with clinical findings suggestive of MAHCM or other conditions associated with a reduced LVOT diameter. The patient’s syncopal episode was likely triggered by dehydration-induced hypovolemia, which exacerbated her underlying diastolic dysfunction and hyperdynamic left ventricular systolic function. Timely intervention with intravenous fluids effectively corrected the AKI and prevented further hemodynamic instability. This case highlights the necessity of evaluating structural cardiomyopathies in patients presenting with unexplained syncope, particularly in the setting of hypovolemia and chronic hypertension. Clinicians should emphasize adequate hydration as a preventive strategy to mitigate syncope risk in susceptible individuals. Further research is warranted to elucidate the precise mechanisms by which hypovolemia contributes to syncope in MAHCM and to optimize individualized management strategies for these patients.
